# Genomic and Transcriptomic Analyses Reveal Pathways and Genes Associated With Brittle Stalk Phenotype in Maize

**DOI:** 10.3389/fpls.2022.849421

**Published:** 2022-04-25

**Authors:** Jun Liu, Chuanbo Sun, Siqi Guo, Xiaohong Yin, Yuling Yuan, Bing Fan, Qingxue Lv, Xinru Cai, Yi Zhong, Yuanfeng Xia, Xiaomei Dong, Zhifu Guo, Guangshu Song, Wei Huang

**Affiliations:** ^1^Maize Research Institute, Jilin Academy of Agricultural Sciences, Gongzhuling, China; ^2^College of Biosciences and Biotechnology, Shenyang Agricultural University, Shenyang, China; ^3^Hulun Buir Agricultural Reclamation Technology Development Co., Ltd., Hailar, China

**Keywords:** maize, brittle stalk, cellulose and lignin synthesis, RNA-seq, BSA-seq

## Abstract

The mechanical strength of the stalk affects the lodging resistance and digestibility of the stalk in maize. The molecular mechanisms regulating the brittleness of stalks in maize remain undefined. In this study, we constructed the maize brittle stalk mutant (*bk5*) by crossing the W22:Mu line with the Zheng 58 line. The brittle phenotype of the mutant *bk5* existed in all of the plant organs after the five-leaf stage. Compared to wild-type (WT) plants, the sclerenchyma cells of *bk5* stalks had a looser cell arrangement and thinner cell wall. Determination of cell wall composition showed that obvious differences in cellulose content, lignin content, starch content, and total soluble sugar were found between *bk5* and WT stalks. Furthermore, we identified 226 differentially expressed genes (DEGs), with 164 genes significantly upregulated and 62 genes significantly downregulated in RNA-seq analysis. Some pathways related to cellulose and lignin synthesis, such as endocytosis and glycosylphosphatidylinositol (GPI)-anchored biosynthesis, were identified by the Kyoto Encyclopedia of Gene and Genomes (KEGG) and gene ontology (GO) analysis. In bulked-segregant sequence analysis (BSA-seq), we detected 2,931,692 high-quality Single Nucleotide Polymorphisms (SNPs) and identified five overlapped regions (11.2 Mb) containing 17 candidate genes with missense mutations or premature termination codons using the SNP-index methods. Some genes were involved in the cellulose synthesis-related genes such as ENTH/ANTH/VHS superfamily protein gene (endocytosis-related gene) and the lignin synthesis-related genes such as the cytochrome p450 gene. Some of these candidate genes identified from BSA-seq also existed with differential expression in RNA-seq analysis. These findings increase our understanding of the molecular mechanisms regulating the brittle stalk phenotype in maize.

## Introduction

As the most produced grain crop, maize (*Zea mays* L.) is an economically important food and forage crop globally. The stalk strength of maize directly influences the lodging resistance and silage quality. Previous studies of maize rinds have indicated that the rind of the stalk is the chief determinant of stalk strength. The structure and composition of the hypodermal cell wall and vascular bundles in the rind directly influence the stalk strength of maize. Furthermore, the stalk strength is strongly associated with the contents and concentration of cellulose, hemicellulose, lignin, and pectin in the cell wall, which directly affect the lodging resistance and digestibility of the stalk (Jiao et al., 2019; Vanholme et al., 2019; Zhang et al., 2019; Liu et al., 2020). To the best of our knowledge, a large number of genes are involved in biosynthesis and modifications of the cell wall (Carpita et al., 2001). Brittle stalk phenotype is also associated with many genes, and only a few studies have explored the functions of the brittle stalk genes. The characterization of mutants is an effective method to identify the functions of brittle stalk genes and individual components of cell walls. Functions of some brittle stalk genes have been characterized using the mutations in the orthologous genes of *Arabidopsis*, rice, maize, wheat, and barley (Kokubo et al., 1989; Li et al., 2003; Ching et al., 2006; Burton et al., 2010; Deng et al., 2019; Jiao et al., 2019).

Cellulose synthase (CESA) genes are strongly related to cell wall synthesis, affecting the mechanical strength of cell walls in plants. Many CESA genes related to brittle stalks, such as *AtCESA1*, *AtCESA3*, *AtCESA4*, *AtCESA7*, and *AtCESA8*, have been identified in *Arabidopsis* (Taylor et al., 2003; Zhong et al., 2003; Persson et al., 2007). Rice mutants corresponding to fragile fiber and collapsed xylem mutants in *Arabidopsis* are brittle culm (*bc*) mutants, displaying a *bc* phenotype (Li et al., 2003; Tanaka et al., 2003). Some CESA genes, such as *OsCESA4*, *OsCESA7*, and *OsCESA9*, have also been studied using the *bc* mutants. The loss-of-function mutations of these genes lead to the reduction of cellulose content and the brittle phenotype (Zhang et al., 2019). COBRA genes encode glycosylphosphatidylinositol (GPI)-anchored proteins (COBRA-like proteins), which influence cellulose biosynthesis in primary and secondary cell walls in plants. The COBRA-like family comprises 12 members in *Arabidopsis* and 11 in rice (Roudier et al., 2002; Li et al., 2003). *AtCOBL4* from *Arabidopsis* and its orthologs, *OsBC1* from rice, *TmBr1* from wheat, and *SbBC1* from sorghum are associated with the mechanical strength of stalks. The loss-of*-*function mutations of these genes also result in the brittle stalk phenotype (Li et al., 2003, 2019; Brown et al., 2005; Persson et al., 2005; Sindhu et al., 2007; Sato et al., 2010; Deng et al., 2019).

As a chitinase-like protein, CTL1 influences cellulose biosynthesis in plants. In *Arabidopsis*, *AtCTL1* plays an important role in establishing interaction between cellulose microfibrils and hemicelluloses, which affects cellulose biosynthesis (Sánchez-Rodríguez et al., 2012). In rice, *OsCTL1* was isolated from the rice *bc15* mutant and found to result in a reduction in mechanical strength and cellulose content (Wu et al., 2012). In addition, some genes that modulate the dynamics and structures of the cytoskeleton influence stalk the brittleness and cell wall biosynthesis. *OsBC12*, an ortholog of the kinesin gene *AtKIF4/FRA1* in *Arabidopsis*, is involved in the microtubule-based effects on cellulose microfibril order. The loss-of-function mutations of *OsBC12* lead to the brittleness phenotype of plants (Zhang et al., 2010). OsBC3, a dynamin-related protein in rice, also affects cellulose biosynthesis and mechanical strength (Xiong et al., 2010).

In maize, some brittle stalk (*bk*) mutants are associated with cellulose biosynthesis and have been utilized to isolate the genes related to brittleness (Langham, 1940; Multani et al., 2003; Ching et al., 2006; Sindhu et al., 2007; Jiao et al., 2019). *ZmBk2* gene identified from *bk2* mutant encodes a COBRA-like protein similar to rice *OsBC1* and *Arabidopsis AtCOBL4*. *ZmBk2* is involved in secondary cellulose biosynthesis of the cell wall, controlling mechanical strength and brittleness of stalks (Sindhu et al., 2007). *ZmCtl1* gene identified from *bk4* mutant encodes a Chitinase-like1 protein, which is expressed at its highest levels in elongated internodes. *ZmCtl1* interacts with CESA proteins, enhancing the tensile strength of stalks (Jiao et al., 2019).

The stalk development of plants is a highly complex process that cannot be completely elucidated by traditional molecular approaches. Although several genes related to brittle stalks have been characterized, the molecular mechanisms regulating the brittleness of stalks in maize remain undefined. It is certain that *the* brittleness phenotype of maize is involved in multiple genes such as rice and *Arabidopsis*. It is absolutely essential to clone more genes related to the mechanical strength and brittleness of stalks using *bk* mutants. As effective methods to identify multiple genes, transcriptome analysis (RNA-seq) and bulked-segregant sequence analysis (BSA-seq) have been widely used to characterize stalk development in various plants. BSA-seq coupled to RNA-seq has also been successfully applied to gene mapping and identification in maize (Liu et al., 2012; Li et al., 2013; Nestler et al., 2014; Tang et al., 2014). In this study, we constructed and identified the maize mutant materials with brittle stalk phenotypes. Cell wall components of the stalk, including cellulose, hemicellulose, lignin, starch, and total soluble sugars, were investigated. Furthermore, some pathways and genes related to brittleness phenotype were identified by RNA-seq combined with BSA-seq. This study provides insight into the molecular basis of the brittle stalk phenotype in maize.

## Materials and Methods

### Plant Material

The maize mutants of the brittle stalk were obtained from the M2 families created by crossing the W22:Mu line (active MuDR donor parent) with the Zheng 58 line (recipient parent). Zheng 58 is the female parent of hybrid Zhengdan958 with high and stable yield, multiresistant, and high plant area in China. To have a uniform background, the material was propagated by backcrossing *bk* mutant with female parent Zheng 58 for additional five times (BC5F1). The BC5F1 was advanced to BC5F2 by selfing for obtaining the mutant (*bk5*) and wild-type (WT; non-brittleness) plants. The mutant *bk5* and WT plants were indistinguishable from each other except for the appearance of a brittleness phenotype at 4 weeks in the mutant.

### Scanning Electron Microscopy

The second internodes of stalks and leaf sections of *bk5* and WT plants in BC5F2 individuals (approximately 1 mm in height) were cut with a razor blade and fixed in 2.5% glutaraldehyde in 0.2 M phosphate buffer (pH = 7.4) overnight at 4°C. After being washed with 0.2 M phosphate buffer, the samples were dried at a critical point under a low vacuum in the desiccators (IXRF, VFD-30, United States) and sputter-coated with gold. All tissues were observed under a Hitachi Regulus 8100 scanning electron microscope (SEM) (Japan) at an acceleration voltage of 10 kV.

### Determination of Stalk Cell Wall Components

The samples were collected from the middle of the second internodes of stalks of *bk5* and non-brittleness plants from BC5F2 individuals in the five-leaf stage and then dried at 105°C for half an hour and 80°C in an oven for 48 h. The samples of total dry mass were ground and screened through a combined 40–80 mesh sieve. The carbohydrates and lignin were extracted using a two-step sulfuric acid hydrolysis process (Hatfield et al., 1994). The sample quantity was measured using a high-performance liquid chromatography (HPLC) system (Agilent 1,100 series). Cellulose content was calculated from the glucose content. Hemicellulose content was determined from the sum of xylose and arabinose contents according to the NREL Laboratory analytical procedure. Lignin content was calculated as the sum of the acid-soluble and acid-insoluble lignin contents (Li et al., 2017). The total soluble sugars were extracted with distilled water and their contents were determined by the anthrone/H_2_SO_4_ method using a UV-vis spectrometer (UV-1800, Shimadzu). Crude starch was purified from stalks by homogenization in 0.7 M perchloric acid and 10 mg of sand. The crude starch pellets with dimethyl sulfoxide were incubated in a boiling water bath for 15 min to disperse the starch. Later, the starch was enzymatically digested, and the content of Glc was determined using a kit from Megazyme (Total Starch Assay Kit, K-TSTA).

### RNA-Seq Analysis

Total RNA was extracted from the stalks of *bk5* and WT plants in BC5F2 individuals after the five-leaf stage using an RNA extraction kit (Aidlab, China) and then treated with RNase-free DNase I (Aidlab, China) to remove genomic DNA. RNA libraries were constructed and sequenced on an Illumina HiSeq 2500 platform to generate 150 bp paired-end reads (Biomarker, China). Raw reads of cDNA fragments were preprocessed to remove adapter and low-quality reads. Eligible reads were aligned to the maize B73 reference genome (AGPv2).^1^

The read numbers mapped to each gene were summarized using HTSeq version 0.6.1 (Anders and Huber, 2010; Hill et al., 2013), and the Fragments PerKilobase Million (FPKM) of each gene was calculated to quantify the gene expression based on read counts mapping to the gene, the length of the gene, and the sequencing depth. The DESeq package in R (version 1.18.0) was used to perform differential expression analysis of the two groups (three biological replicates per group). The *P*-values were adjusted using Benjamini and Hochberg’s method to control for false discovery rates. Genes with an adjusted *p* < 0.05 were set as the criteria to filter the differentially expressed genes (DEGs). Gene ontology (GO) enrichment analysis of DEGs was performed with the GOseq R package (Young et al., 2010). GO terms with a corrected *P*-value of < 0.05 were considered to be significantly enriched in DEGs. The Kyoto Encyclopedia of Genes and Genomes (KEGG) pathway annotations were assigned according to the KEGG database.^2^ The KOBAS software (Mao et al., 2005) was used to test the enrichment of DEGs in KEGG pathways.

### Bulked-Segregant Sequence Analysis

A total of 60 maize plants (B694, 30 brittleness plants, B695, 30 non-brittleness plants) from the above-mentioned BC5F2 population were used for BSA-seq analysis. Total DNA was extracted from leaves with a DNA extraction kit (Aidlab, China), and two DNA pools were constructed by mixing equal amounts of DNA from 30 brittleness and 30 non-brittleness plants. The libraries of these two DNA pools were constructed using an Illumina sequencing platform (HiSeq2500; Illumina). For genomic re-sequencing, the paired-end 100-base (PE100) reads were generated and aligned to the maize B73 reference genome sequences (AGPv2; see text footnote 1) using BWA (Li and Durbin, 2009). The GATK software (McKenna et al., 2010) was used to detect SNPs and small indels and correct for alignment errors. The variants were removed if more than two SNPs occurred in a 5-bp window if the distance was less than 10 bp between two indels or SNPs were within 5 bp of an indel. The association analysis was conducted by the InDel index (Fekih et al., 2013).

### Real-Time Quantitative PCR

The real-time quantitative PCR (qRT-PCR) was performed to identify the expression patterns shown by the RNA-seq analysis. Fourteen genes were randomly selected on the basis of their potential functions in RNA-seq. The qRT-PCR was carried out with a Monad Selected q225 Real-Time PCR System (Monad, China) under the following conditions: 95°C for 3 min; 35 cycles of 95°C for 5 s, 58°C for 30 s, and 72°C for 2 min; and 1 cycle of 72°C for 10 min. Each sample was analyzed in the three biological and three technical replicates. The relative expression levels were calculated *via* the ΔΔCt method (Livak and Schmittgen, 2001). The actin gene of maize (GenBank accession no. AY273142) was used as a reference gene. All primer sequences are listed in Supplementary Table 1.

### Statistical Analysis

All data that needed statistical analysis are shown as mean values ± SE of the mean by using 3 replicates. The SPSS Statistics^3^ software was used for statistical analysis *via* Student’s *t*-test. Significant differences were considered significant with a probability level of *p* < 0.05.

## Results

### Phenotypic Characteristics of the Brittle Stalk Mutant *bk5*

**FIGURE 1:**
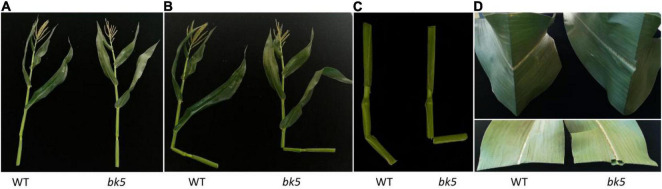
Phenotypes of stalks and leaves of wild-type (WT) and mutant plants at the stage of anthesis. **(A)** Normal growth conditions. **(B)** Broken stalks. **(C)** Enlargement of broken stalks. **(D)** Broken leaves.

In the uniform backcross progeny, the mutants were phenotypically indistinguishable from WT siblings, except that the stalk and leaves of mutants were brittle after the five-leaf stage throughout the life of the plant (Figure 1). To confirm whether known genes result in the mutant phenotype, we identified the sequences of brittle stalk genes *ZmBk2* and *ZMBk4* in the mutants and WT using PCR sequencing. No sequence difference was found in these two genes and their promoters between the mutant and WT (Supplementary Figure 1), indicating that some other genes or factors except for *ZmBk2* and *ZMBk4* could lead to the brittleness phenotype in the mutants. Thus, this mutant was named *bk5* for further investigation.

### Determination of Cell Structure and Cell Wall Composition

**FIGURE 2:**
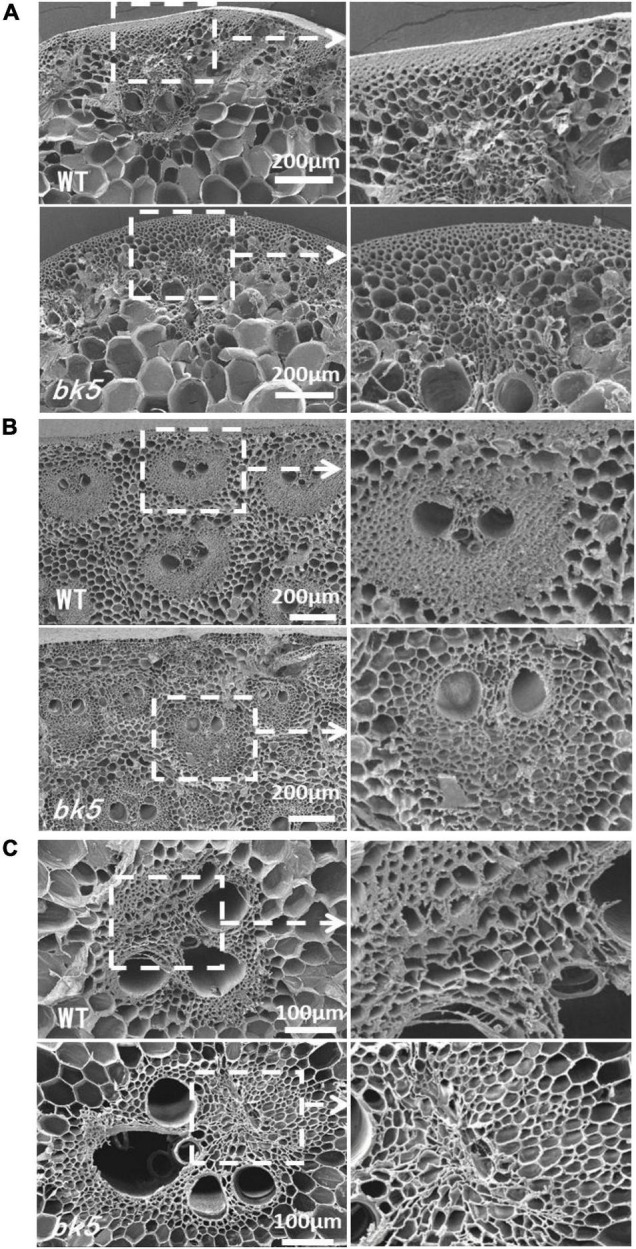
Scanning electron micrographs of *bk5* and WT stems and veins taken from cross sections. **(A)** Leaf veins and local enlargement. **(B)** Rind regions of the stalk and local enlargement of the vascular bundle. **(C)** Central regions of the stalk and local enlargement of vascular bundle.

To determine whether the brittleness phenotype of *bk5* arises from changes in cell structure, we compared the sclerenchyma cells of internode stalks and leaf veins of *bk5* with WT siblings using SEM. Compared to WT plants, the sclerenchyma cells in leaf veins (Figure 2A) and stalks (Figures 2B,C) of *bk5* had a looser arrangement. The sclerenchyma cell wall was thinner in the rind region and the stalk vascular bundles in *bk5* than those of WT plants (Figure 2). These results demonstrated that the thickness of cell walls and the tightness of cell–cell junctions directly affected the stalk brittleness.

**FIGURE 3:**
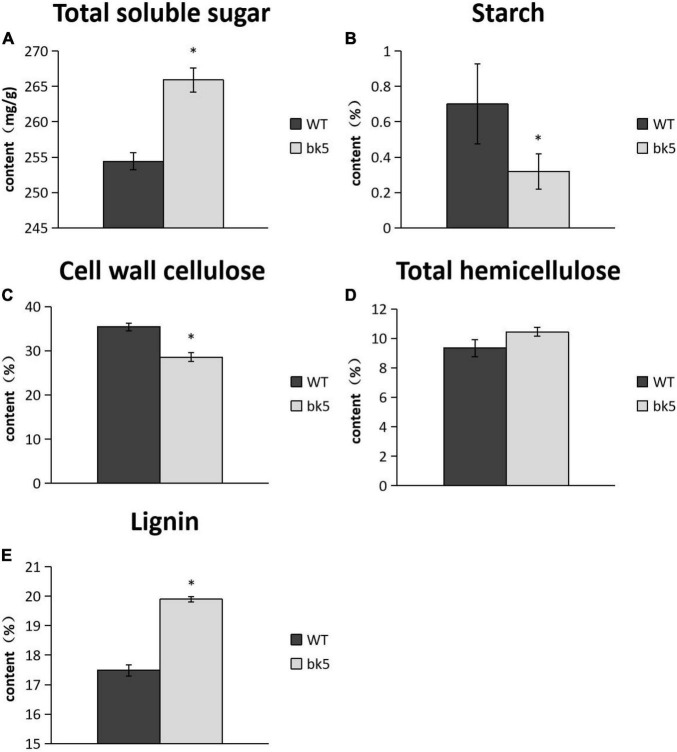
Determination of cell wall composition. **(A)** Content of total soluble sugar. **(B)** Starch Content. **(C)** Cell wall cellulose. **(D)** Total hemicellulose content. **(E)** Lignin content. Biological triplicates were averaged and statistically analyzed *via* Student’s *t*-test (*p < 0.05).

To further explore the association between brittleness and cellular components, we analyzed the cell wall composition of stalks in *bk5* and WT plants. The results showed that the cellulose content in cell walls and the starch content in *bk5* stalks were significantly lower than those of WT plants (Figures 3B,C), while total soluble sugar content and lignin content were significantly higher than those of WT plants (Figures 3A,E). No obvious difference in hemicellulose content was found between *bk5* and WT plants (Figure 3D). These results indicated that the cellulose and starch contents in the cell walls of maize stalks are directly related to the stalk brittleness.

### RNA-Seq Analysis

#### Comparison of Gene Expression Between the Mutant *bk5* and Wild-Type

**FIGURE 4:**
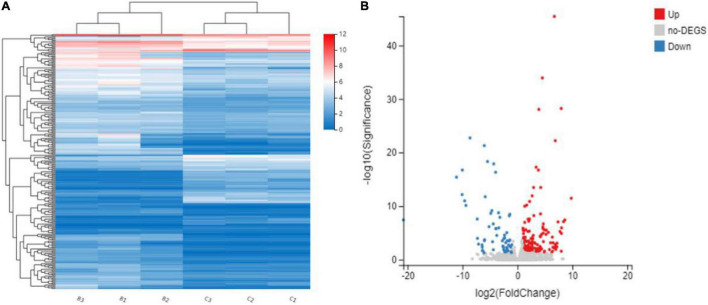
Hierarchical cluster analysis **(A)** and volcano plots **(B)** of differentially expressed genes (DEGs) between mutant *bk5* and WT. Averaged log_2_ relative NRPKM of all the genes in each cluster was used to generate the heat map.

**FIGURE 5:**
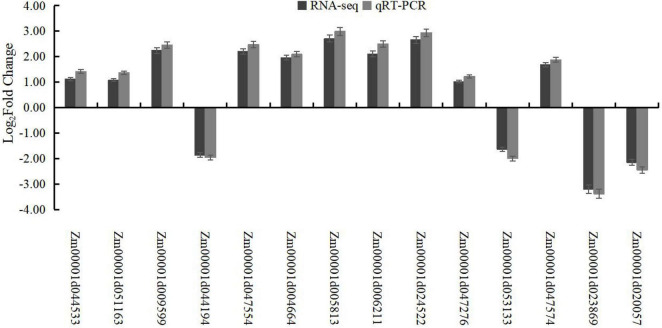
Validation of RNA-seq expression by qRT-PCR. The actin gene (GenBank accession no. AY273142) was used as an internal control.

To investigate the transcriptomic difference between *bk5* and WT, RNA-seq analysis was performed. In total, 49.34–65.74 million clean reads were generated for each sample. After the removal of low-quality reads, duplicated sequences, adaptor sequences, and ambiguous reads, 78.18–87.14% of reads were uniquely mapped to the Maize B73 reference genome (Table 1). DEGs were compared between the *bk5* and WT stalks, and the expression patterns of DEGs were also confirmed by hierarchical clustering to identify the groups of different biological replicates with similar expression patterns (Figure 4A). Comparison results showed that 226 DEGs were detected, with 164 genes significantly upregulated and 62 genes significantly downregulated (Figure 4B and Supplementary Table 2). In these DEGs, *ZmBk2* was also detected (Figure 5, Zm00001d047276), indicating that the expression of *ZmBk2* may be affected by some factors.

**TABLE 1:** Summary of sequencing and mapping from *bk5* and WT plants for RNA-seq analysis.

Sample	Total clean reads (M)	Total clean bases (Gb)	Total mapping (%)	Unique mapping (%)
B1	65.74	9.82	90.58	87.14
B2	58.69	8.76	84.55	80.75
B3	63.72	9.52	91.5	87.93
C1	59.41	8.87	81.26	78.18
C2	57.94	8.65	89.79	86.31
C3	49.34	7.36	86.56	82.98

#### Gene Ontology and Kyoto Encyclopedia of Genes and Genomes Analyses of Differentially Expressed Genes in Brittle Stalk Development

**FIGURE 6:**
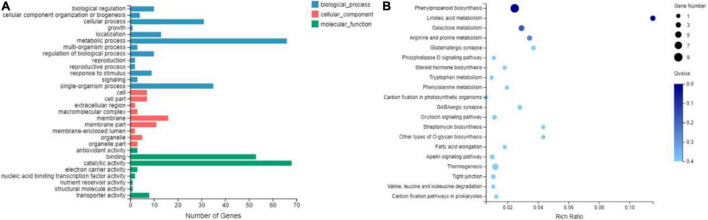
Gene ontology classification **(A)** and enriched Kyoto Encyclopedia of Genes and Genome pathway scatterplots **(B)** of DEGs shared by mutant *bk5* vs. WT.

The GO enrichment analysis was performed to classify the functions of all DEGs. A significantly enriched GO term was identified with the corrected *P* < 0.05. The top 30 significant subcategories were finally enriched for GO functional categories, and 13, 9, and 8 functional GO terms were identified for biological processes, cellular components, and molecular functions, respectively (Figure 6A). For example, “metabolic process” (GO:0008152, 66 genes) in the biological processes, “catalytic activity” (GO:0003824, 68 genes) and “binding” (GO:0005488, 53 genes) in the molecular function category was the most highly enriched GO terms. Furthermore, the KEGG enrichment analysis of DEGs was performed to identify the biological pathways. The top 20 KEGG pathways with the most representation were shown in Figure 6B. Many pathways were involved in the cellulose synthesis*-*related pathways, such as endocytosis (Ko04144), GPI-anchored biosynthesis (Ko00563), tryptophan metabolism (Ko00380), glycerolipid metabolism (Ko00561), and calcium signaling pathway (Ko04020), as well as lignin synthesis*-*related pathways, such as steroid hormone biosynthesis (Ko00140) and phenylalanine metabolism (Ko00360). In addition, the starch and sucrose metabolism (Ko00500) pathways were also enriched. The expression levels of the genes related to these pathways in RNA-seq have been listed in Supplementary Table 3. These pathways are probably associated with the brittleness phenotype in maize.

#### Validation of RNA-Seq Data by Quantitative PCR

To confirm the accuracy of transcriptome sequencing, 14 genes, including 5 genes related to cellulose synthesis, lignin synthesis, and starch and sucrose metabolism*-*related pathways, were selected, and their expression profiles were analyzed using qRT-PCR. The results showed the same expression profiles between qRT-PCR assays and RNA-seq analysis, which showed the high reliability of transcriptome sequencing (Figure 5).

### Bulked-Segregant Sequence Analysis

**FIGURE 7:**
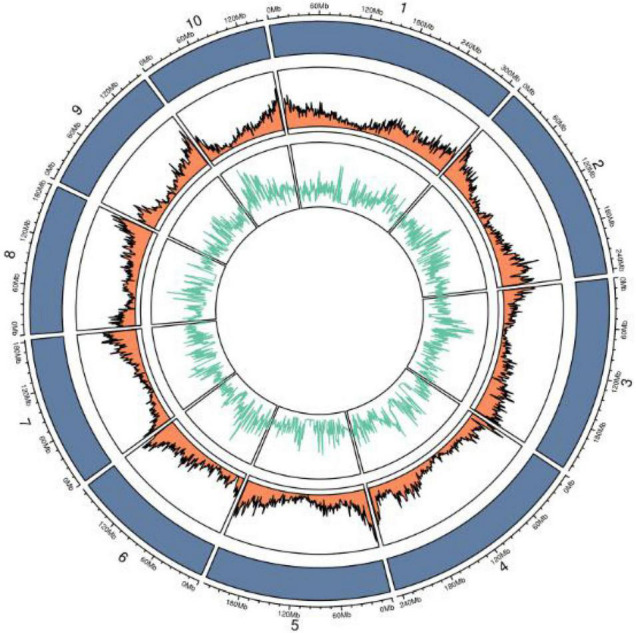
Circos diagram of SNPs on different chromosomes.

Whole-genome re-sequencing data were generated for brittleness bulk (B694) and non-brittleness bulk (B695) using the Illumina HiSeq2500. In total, 69.55 million clean reads were generated for the brittleness bulk and 49.57 million clean reads for the non-brittleness bulk. Mapping of those reads to the reference genome B73 led to 52.16X and 37.17X coverage with 86.78 and 86.11% mapping efficiency for brittleness and non-brittleness bulks, respectively. The obtained sequencing data were 10.43 GB for B694 and 7.44 GB for B695 (Table 2). Then, 2,931,692 high-quality SNPs were detected after filtering SNPs with low coverage and discrepancy (Figure 7).

**TABLE 2:** Summary of sequencing and mapping from brittleness bulk (B694) and non-brittleness bulk (B695) for BSA-seq analysis.

Sample	Clean reads	Mapped read	Mapping efficiency (%)	Average depth (X)	Data generated (Gb)
B694	695,467,028	603,510,435	86.78	52.16	10.43
B695	495,658,970	426,803,758	86.11	37.17	7.44

**FIGURE 8:**
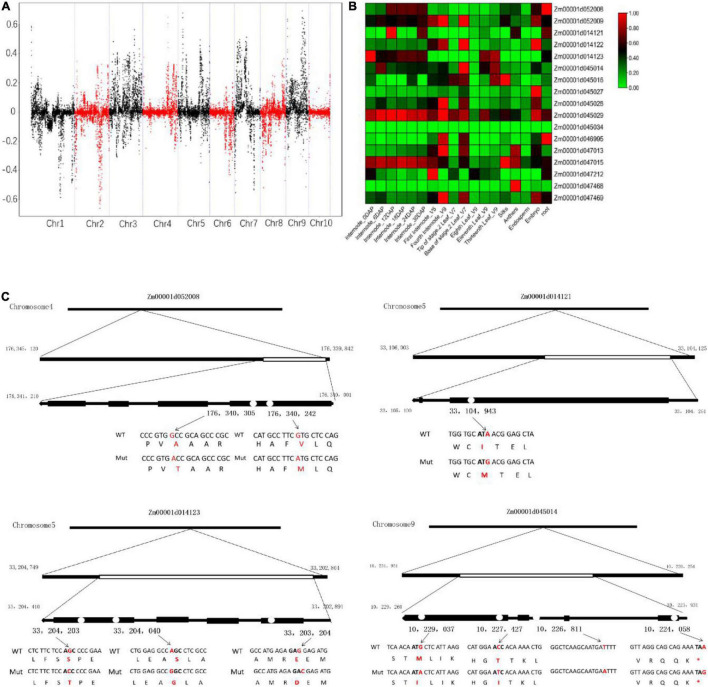
Allele frequency difference graph from bulked-segregant sequence analysis **(A)**, heat map of expression profiles of candidate genes in maize **(B)**, and the sequence variations of the candidate genes related to the cellulose and lignin synthesis **(C)**. The standard deviations for the genome-wide median were chosen based on the threshold value of the confidence interval of 0.6.

To examine the genomic region related to brittleness phenotype, the SNP-index methods were used to detect the allele segregation of SNPs between the two bulks. The SDs for the genome-wide median were chosen based on the threshold value of the confidence interval of 0.6. Finally, we identified five overlapped regions containing 17 candidate genes with missense mutations or premature termination codons. These genes were located on chromosome 4 (0.3 Mb region containing 2 genes), chromosome 5 (0.2 Mb region containing 3 genes), and chromosome 9 (10.7 Mb containing 12 genes), respectively (Figure 8A and Table 3). Some of these genes were involved in the cellulose synthesis such as ENTH/ANTH/VHS superfamily protein gene (Zm00001d052008, endocytosis-related gene) and chloroplast RNA splicing-related gene (Zm00001d045014), as well as the lignin synthesis such as cytochrome p450 (Zm00001d014121) and peroxidase superfamily protein gene (Zm00001d014123). The expression levels of some candidate genes were different in various tissues and at different growth stages of the same tissue, especially the internode of the stalk (Figure 8B). In addition, some candidate genes identified from BSA-seq also existed with differential expression in RNA-seq analysis. Furthermore, the sequence variations of the above-mentioned candidate genes were analyzed. These sequence variations from SNP sites mainly resulted due to some changes in amino acid residues (Figure 8C). This needs further study on whether the Mu transposons cause these sequence variations.

**TABLE 3:** Candidate genes for brittleness phenotype based on the SNP-index methods.

Gene ID	Location of genes	Gene annotation	ID of homologous genes in *Arabidopsis*
Zm00001d052008	Chr4:176339769–176344984	ENTH/ANTH/VHS superfamily protein	AT5G35200.1
Zm00001d052009	Chr4:176346608–176364635	MAK10 homolog	AT2G11000.1
Zm00001d014121	Chr5:33104080–33106019	Cytochrome p450, family 71, subfamily B, polypeptide 11	AT5G25120.1
Zm00001d014122	Chr5:33167513–33179297	Translin family protein	AT2G37020.2
Zm00001d014123	Chr5:33202990–33204555	Peroxidase superfamily protein	AT5G19890.1
Zm00001d045014	Chr9:10221137–10230897	Chloroplast RNA splicing3	
Zm00001d045016	Chr9:10280560–10281964	Unknown	
Zm00001d045027	Chr9:10607821–10611813	3-ketoacyl-acyl carrier protein synthase I	AT5G46290.1
Zm00001d045028	Chr9:10620254–10622266	Nucleolar GTP-binding protein	AT1G50920.1
Zm00001d045029	Chr9:10624258–10628083	MITOCHONDRIALFERREDOXIN 2	AT4G21090.1
Zm00001d045034	Chr9:10656402–10661489	Unknown	
Zm00001d046995	Chr9:114408410–114410512	Patellin-5	
Zm00001d047013	Chr9:115242444–115244729	Entatricopeptide repeat-containing protein mitochondrial	
Zm00001d047015	Chr9:115397715–115419683	DNAJ heat shock N-terminal domain-containing protein	AT2G26890.1
Zm00001d047212	Chr9:122291025–122292698	Metacaspase 1	AT1G02170.1
Zm00001d047468	Chr9:131003201–131026782	Golgi-body localization protein domain; RNA pol II promoter Fmp27 protein domain	AT1G58250.1
Zm00001d047469	Chr9:131085527–131087944	Putative nucleolar-protein-domain family protein	AT1G11240.1

## Discussion

As a complex trait, the stalk development in plants relates closely to many biological processes, including cell wall synthesis, cell wall modification, and vascular bundle formation. These factors directly affect the mechanical strength of the stalk. It was confirmed that the brittle stalk phenotype is associated with many genes (Roudier et al., 2002; Li et al., 2003). In many plants, some genes related to the brittleness phenotype have been identified, while more than one brittle stalk gene was cloned in maize (Sindhu et al., 2007; Jiao et al., 2019). Thus, identifying more brittle stalk genes is important for understanding the molecular mechanism of the stalk’s mechanical strength and brittleness in plants. Because of the fragile stalk strength, the brittle stalk trait in crops is clearly unfavorable for lodging resistance. However, reducing the content of cellulose and lignin can increase the digestibility of the stalk, which increases the feed value of crops, especially maize silage (Wang et al., 2020; Vinayan et al., 2021). From this perspective, the brittle stalk trait in crops is favorable for the feed industry. For breeding applications aiming at unfavorable or favorable traits, molecular markers based on brittle stalk genes can be developed to assist maize breeding.

Published studies have shown that the brittle stalk phenotype in plants is strongly associated with cellulose synthesis and lignin synthesis (Jiao et al., 2019). As the most abundant polysaccharide in plant cell walls, cellulose is synthesized at the plasma membrane by CESA complexes (CSC) using uridine diphosphate (UDP)-glucose as substrates (Somerville, 2006). In this process, the transport of CSCs from the Golgi to the plasma membrane, UDP-glucose, and endocytosis is very vital for cellulose biosynthesis. The trans-Golgi network/early endosome (TGN/EE) compartment has been implicated in protein sorting and as a hub of plant exocytosis and endocytotic pathways. The Golgi-localized STELLO1 and 2 (STL1/2) proteins regulate cellulose synthesis likely by controlling the assembly of CESA CSCs in the Golgi (Zhang et al., 2016; Polko and Kieber, 2019). In this study, an endocytosis-related gene (Zm00001d052008), ENTH/ANTH/VHS superfamily protein gene was identified in BSA-seq analysis. Coincidentally, the endocytosis pathway (Ko04144) was also enriched in the KEGG analysis of RNA-seq. It was shown that the endocytosis may be associated with the brittle stalk phenotype. In addition, a molecular function category, UDP-galactosyltransferase activity (GO:0035250), was detected in GO enrichment analysis.

Besides the CSC, cellulose synthesis is also highly related to other factors such as transcription factors, COBRA-like protein, tryptophan metabolism, etc. (Bray et al., 1996; Dai et al., 2011; Noda et al., 2015). The transcription factor OsMYB103 is a transcriptional regulator of cellulose synthesis by targeting CESA genes in rice (Yang et al., 2014). Some NAC transcription factors are transcriptional switches for the difference of xylem vessels, which regulate the cellulose synthesis and the formation of secondary cell walls in the tissues (Kubo et al., 2005). In this study, the expression levels of MYB-related-transcription factor genes (Zm00001d044194 and Zm00001d009599) and NAC-transcription factor 81 gene (Zm00001d047554) differed between *bk5* and WT based on the RNA-seq analysis. These transcription factors may regulate the expression of some genes related to cellulose synthesis in maize, which affects the brittle stalk phenotype. As previously mentioned, *ZmBk2* encodes a COBRA-like protein (GPI-anchored protein), which results in the brittleness phenotype in maize mutant *bk2* (Sindhu et al., 2007). In this study, we did not find the sequence difference of *ZmBk2* and its promoter between *bk5* and WT by BSA-seq and PCR sequencing. However, the expression difference of *ZmBk2* between *bk5* and WT was found by the RNA-seq analysis. In the KEGG analysis, the GPI-anchored biosynthesis pathway (Ko00563) was also enriched. These results indicated that the expression of *ZmBk2* may be affected by more genes or some unknown factor(s). Tryptophan oxidation can reduce the extent of binding of the cellulose-binding domain of an exoglucanase/xylanase (CBDcex) to cellulose, which affects the mechanical strength of the stalk in plants (Bray et al., 1996). In this study, the tryptophan metabolism pathway (Ko00380) was enriched in the KEGG analysis. Taken together, these genes or pathways may regulate the cellulose synthesis, which in turn lead to the brittle stalk phenotype in maize. Naturally, more work is required to elaborate on these speculations.

Besides cellulose synthesis, lignin is also associated with the mechanical strength of the stalk in maize. In rice *bc* mutant *bc1*, sorghum *bc* mutant *sbbc1*, and maize brittle stalk mutant *bk2*, the lignin contents were higher than those of WT. Furthermore, it was speculated that patterning of lignin–cellulose interactions resulted in the brittle stalk phenotype of mutant *bk2* (Li et al., 2003, 2019; Ching et al., 2006; Sindhu et al., 2007). In addition, the lignin synthesis relates to some secondary metabolites and aromatic compounds. For example, as a complex biopolymer, the lignin crosslinks with the phenylpropanoid units in plant secondary cell walls. The lignin biosynthesis requires the cytochrome P450 enzymes located in the endoplasmic reticulum to establish the structural characteristics of its monomeric precursors (Gou et al., 2018; Morgan et al., 2019). In addition, phenylalanine, tyrosine, and phenylpropane biosynthesis pathways also affect lignin synthesis (Liu et al., 2018). In this study, the lignin content of mutant *bk5* was also higher than that of WT. In BSA-seq analysis, we identified a gene coding a polypeptide 11 in subfamily B of Cytochrome p450 (Zm00001d014121), while a phenylalanine metabolism (Ko00360) and a cellular aromatic compound metabolic process (GO:0006725) were enriched in KEGG analysis and GO enrichment analysis in RNA-seq, respectively. These pathways or corresponding genes may be associated with the lignin synthesis, which in turn affect the brittle stalk phenotype of maize. Certainly, further investigations are required to shed light on some key critical issues. In conclusion, some genes and metabolism pathways possibly related to the brittle stalk phenotype were identified by RNA-seq and BSA-seq analysis, which provide an important reference for further understanding of the molecular mechanisms regulating the brittle stalk phenotype in maize.

## Data Availability Statement

The datasets presented in this study can be found in online repositories. The name of the repository and accession number(s) can be found below: National Center for Biotechnology Information (NCBI) BioProject, https://www.ncbi.nlm.nih.gov/bioproject/, PRJNA794286 and PRJNA794303.

## Author Contributions

WH and GS designed and supervised the study. JL, CS, XY, YY, and BF constructed the materials. JL, SG, and QL determined phenotypic data and cell wall composition. JL, CS, XC, and SG performed the BSA-seq and RNA-seq analysis. JL, YZ, and YX performed RNA isolation and qRT-PCR experiments. ZG and XD contributed to revising the manuscript. All authors contributed to the article and approved the submitted version.

## Conflict of Interest

YY and BF were employed by the company Hulun Buir Agricultural Reclamation Technology Development Co., Ltd. The remaining authors declare that the research was conducted in the absence of any commercial or financial relationships that could be construed as a potential conflict of interest.

## Publisher’s Note

All claims expressed in this article are solely those of the authors and do not necessarily represent those of their affiliated organizations, or those of the publisher, the editors and the reviewers. Any product that may be evaluated in this article, or claim that may be made by its manufacturer, is not guaranteed or endorsed by the publisher.

## Footnotes

1
http://ftp.maizesequence.org/

2
http://www.genome.jp/kegg

3
www.ibm.com/products/spss-statistics

## References

[B1] Anders S. Huber W. (2010). Differential expression analysis for sequence count data. *Genome Biol*. 11:R106. 10.1186/gb-2010-11-10-r106 20979621 PMC3218662

[B2] Bray M. R. Johnson P. E. Gilkes N. R. McIntosh L. P. Kilburn D. G. Warren R. A. (1996). Probing the role of tryptophan residues in a cellulose-binding domain by chemical modification. *Protein Sci*. 5 2311–2318. 10.1002/pro.5560051117 8931149 PMC2143281

[B3] Brown D. M. Zeef L. A. Ellis J. Goodacre R. Turner S. R. (2005). Identification of novel genes in Arabidopsis involved in secondary cell wall formation using expression profiling and reverse genetics. *Plant Cell* 17 2281–2295. 10.1105/tpc.105.031542 15980264 PMC1182489

[B4] Burton R. A. Ma G. Baumann U. Harvey A. J. Shirley N. J. Taylor J. (2010). A customized gene expression microarray reveals that the brittle stem phenotype fs2 of barley is attributable to a retroelement in the *HvCesA4* cellulose synthase gene. *Plant Physiol*. 153 1716–1728. 10.1104/pp.110.158329 20530215 PMC2923883

[B5] Carpita N. C. Defernez M. Findlay K. Wells B. Shoue D. A. Catchpole G. (2001). Cell wall architecture of the elongating maize coleoptile. *Plant Physiol.* 127 551–565. 10.1104/pp.010146 11598229 PMC125090

[B6] Ching A. Dhugga K. S. Appenzeller L. Meeley R. Bourett T. M. Howard R. J. (2006). Brittle stalk 2 encodes a putative glycosylphosphatidylinositol-anchored protein that affects mechanical strength of maize tissues by altering the composition and structure of secondary cell walls. *Planta* 224 1174–1184. 10.1007/s00425-006-0299-8 16752131

[B7] Dai X. You C. Chen G. Li X. Zhang Q. Wu C. (2011). OsBC1L4 encodes a COBRA-like protein that affects cellulose synthesis in rice. *Plant Mol. Biol*. 75 333–345. 10.1007/s11103-011-9730-z 21264494

[B8] Deng Q. Kong Z. Wu X. Ma S. Yuan Y. Jia H. (2019). Cloning of a COBL gene determining brittleness in diploid wheat using a MapRseq approach. *Plant Sci*. 285 141–150. 10.1016/j.plantsci.2019.05.011 31203879

[B9] Fekih R. Takagi H. Tamiru M. Abe A. Natsume S. Yaegashi H. (2013). MutMap+: genetic mapping and mutant identification without crossing in rice. *PLoS One* 8:e68529. 10.1371/journal.pone.0068529 23874658 PMC3707850

[B10] Gou M. Ran X. Martin D. W. Liu C. J. (2018). The scaffold proteins of lignin biosynthetic cytochrome P450 enzymes. *Nat. Plants* 4 299–310. 10.1038/s41477-018-0142-9 29725099

[B11] Hatfield R. D. Jung H.-J. G. Ralph J. Buxton D. R. Weimer P. J. (1994). A comparison of the insoluble residues produced by the Klason lignin and acid detergent lignin procedures. *J. Sci. Food Agric.* 65 51–58. 10.1002/jsfa.2740650109

[B12] Hill J. T. Demarest B. L. Bisgrove B. W. Gorsi B. Su Y. C. Yost H. J. (2013). MMAPPR: mutation mapping analysis pipeline for pooled RNA-seq. *Genome Res*. 23 687–697. 10.1101/gr.146936.112 23299975 PMC3613585

[B13] Jiao S. Hazebroek J. P. Chamberlin M. A. Perkins M. Sandhu A. S. Gupta R. (2019). Chitinase-like1 plays a role in stalk tensile strength in maize. *Plant Physiol*. 181 1127–1147. 10.1104/pp.19.00615 31492738 PMC6836851

[B14] Kokubo A. Kuraishi S. Sakurai N. (1989). Culm strength of barley: correlation among maximum bending stress, cell wall dimensions, and cellulose content. *Plant Physiol*. 91 876–882. 10.1104/pp.91.3.876 16667151 PMC1062090

[B15] Kubo M. Udagawa M. Nishikubo N. Horiguchi G. Yamaguchi M. Ito J. (2005). Transcription switches for protoxylem and metaxylem vessel formation. *Genes Dev*. 19 1855–1860. 10.1101/gad.1331305 16103214 PMC1186185

[B16] Langham D. G. (1940) The inheritance of intergeneric differences in zea-euchlaena hybrids. *Genetics* 25, 88–107. 10.1093/genetics/25.1.88 17246959 PMC1209080

[B17] Li H. Durbin R. (2009). Fast and accurate short read alignment with Burrows-Wheeler transform. *Bioinformatics* 25 1754–1760. 10.1093/bioinformatics/btp324 19451168 PMC2705234

[B18] Li K. Wang H. Hu X. Ma F. Wu Y. Wang Q. (2017). Genetic and quantitative trait locus analysis of cell wall components and forage digestibility in the zheng58 × HD568 maize RIL population at anthesis stage. *Front. Plant Sci*. 8:1472. 10.3389/fpls.2017.01472 28883827 PMC5573715

[B19] Li P. Liu Y. Tan W. Chen J. Zhu M. Lv Y. (2019). Brittle culm 1 encodes a COBRA-like protein involved in secondary cell wall cellulose biosynthesis in sorghum. *Plant Cell Physiol*. 60 788–801. 10.1093/pcp/pcy246 30590744

[B20] Li S. Ge F. R. Xu M. Zhao X. Y. Huang G. Q. Zhou L. Z. (2013). *Arabidopsis* COBRA-LIKE 10, a GPI-anchored protein, mediates directional growth of pollen tubes. *Plant J.* 74 486–497. 10.1111/tpj.12139 23384085

[B21] Li Y. Qian Q. Zhou Y. Yan M. Sun L. Zhang M. (2003). BRITTLE CULM1, which encodes a COBRA-like protein, affects the mechanical properties of rice plants. *Plant Cell* 15 2020–2031. 10.1105/tpc.011775 12953108 PMC181328

[B22] Liu F. Ahmed Z. Lee E. A. Donner E. Liu Q. Ahmed R. (2012). Allelic variants of the amylose extender mutation of maize demonstrate phenotypic variation in starch structure resulting from modified protein-protein interactions. *J. Exp. Bot.* 63 1167–1183. 10.1093/jxb/err341 22121198 PMC3276085

[B23] Liu Q. Luo L. Zheng L. (2018). Lignins: biosynthesis and biological functions in plants. *Int. J. Mol. Sci.* 19:335. 10.3390/ijms19020335 29364145 PMC5855557

[B24] Liu X. Hu X. Li K. Liu Z. Wu Y. Wang H. (2020). Genetic mapping and genomic selection for maize stalk strength. *BMC Plant Biol*. 20:196. 10.1186/s12870-020-2270-4 32380944 PMC7204062

[B25] Livak K. J. Schmittgen T. D. (2001). Analysis of relative gene expression data using real-time quantitative pcr. *Methods* 25 402–408. 10.1006/meth.2001.1262 11846609

[B26] Mao X. Cai T. Olyarchuk J. G. Wei L. (2005). Automated genome annotation and pathway identification using the KEGG Orthology (KO) as a controlled vocabulary. *Bioinformatics* 21 3787–3793. 10.1093/bioinformatics/bti430 15817693

[B27] McKenna A. Hanna M. Banks E. Sivachenko A. Cibulskis K. Kernytsky A. (2010). The genome analysis toolkit: a mapreduce framework for analyzing next-generation DNA sequencing data. *Genome Res*. 20 1297–1303. 10.1101/gr.107524.110 20644199 PMC2928508

[B28] Morgan K. Martucci N. Kozlowska A. Gamal W. Brzeszczyński F. Treskes P. (2019). Chlorpromazine toxicity is associated with disruption of cell membrane integrity and initiation of a pro-inflammatory response in the HepaRG hepatic cell line. *Biomed. Pharmacother.* 111, 1408–1416. 10.1016/j.biopha.2019.01.020 30841456

[B29] Multani D. S. Briggs S. P. Chamberlin M. A. Blakeslee J. J. Murphy A. S. Johal G. S. (2003). Loss of an MDR transporter in compact stalks of maize br2 and sorghum dw3 mutants. *Science* 302 81–84. 10.1126/science.1086072 14526073

[B30] Nestler J. Liu S. Wen T. J. Paschold A. Marcon C. Tang H. M. (2014). Roothairless5, which functions in maize (*Zea mays* L.) root hair initiation and elongation encodes a monocot-specific NADPH oxidase. *Plant J.* 79 729–740. 10.1111/tpj.12578 24902980

[B31] Noda S. Koshiba T. Hattori T. Yamaguchi M. Suzuki S. Umezawa T. (2015). The expression of a rice secondary wall-specific cellulose synthase gene, OsCesA7, is directly regulated by a rice transcription factor, OsMYB58/63. *Planta* 242 589–600. 10.1007/s00425-015-2343-z 26070439

[B32] Persson S. Paredez A. Carroll A. Palsdottir H. Doblin M. Poindexter P. (2007). Genetic evidence for three unique components in primary cell-wall cellulose synthase complexes in *Arabidopsis*. *Proc. Natl. Acad. Sci. U.S.A.* 104 15566–15571. 10.1073/pnas.0706592104 17878302 PMC2000526

[B33] Persson S. Wei H. Milne J. Page G. P. Somerville C. R. (2005). Identification of genes required for cellulose synthesis by regression analysis of public microarray data sets. *Proc. Natl. Acad. Sci. U.S.A.* 102 8633–8638. 10.1073/pnas.0503392102 15932943 PMC1142401

[B34] Polko J. K. Kieber J. J. (2019). The regulation of cellulose biosynthesis in plants. *Plant Cell* 31 282–296. 10.1105/tpc.18.00760 30647077 PMC6447023

[B35] Roudier F. Schindelman G. DeSalle R. Benfey P. N. (2002). The COBRA family of putative GPI-anchored proteins in Arabidopsis. A new fellowship in expansion. *Plant Physiol*. 130 538–548. 10.1104/pp.007468 12376623 PMC166585

[B36] Sánchez-Rodríguez C. Bauer S. Hématy K. Saxe F. Ibáñez A. B. Vodermaier V. (2012). Chitinase-like1/pom-pom1 and its homolog CTL2 are glucan-interacting proteins important for cellulose biosynthesis in *Arabidopsis*. *Plant Cell* 24 589–607. 10.1105/tpc.111.094672 22327741 PMC3315235

[B37] Sato K. Ito S. Fujii T. Suzuki R. Takenouchi S. Nakaba S. (2010). The carbohydrate-binding module (CBM)-like sequence is crucial for rice CWA1/BC1 function in proper assembly of secondary cell wall materials. *Plant Signal. Behav.* 5 1433–1436. 10.4161/psb.5.11.13342 21051956 PMC3115247

[B38] Sindhu A. Langewisch T. Olek A. Multani D. S. McCann M. C. Vermerris W. (2007). Maize brittle stalk2 encodes a COBRA-like protein expressed in early organ development but required for tissue flexibility at maturity. *Plant Physiol*. 145 1444–1459. 10.1104/pp.107.102582 17932309 PMC2151699

[B39] Somerville C. (2006). Cellulose synthesis in higher plants. *Annu. Rev. Cell Dev. Biol.* 22 53–78. 10.1146/annurev.cellbio.22.022206.160206 16824006

[B40] Tanaka K. Murata K. Yamazaki M. Onosato K. Miyao A. Hirochika H. (2003). Three distinct rice cellulose synthase catalytic subunit genes required for cellulose synthesis in the secondary wall. *Plant Physiol*. 133 73–83. 10.1104/pp.103.022442 12970476 PMC196581

[B41] Tang H. M. Liu S. Hill-Skinner S. Wu W. Reed D. Yeh C. T. (2014). The maize brown midrib2 (bm2) gene encodes a methylenetetrahydrofolate reductase that contributes to lignin accumulation. *Plant J.* 77 380–392. 10.1111/tpj.12394 24286468 PMC4282534

[B42] Taylor N. G. Howells R. M. Huttly A. K. Vickers K. Turner S. R. (2003). Interactions among three distinct CesA proteins essential for cellulose synthesis. *Proc. Natl. Acad. Sci. U.S.A.* 100 1450–1455. 10.1073/pnas.0337628100 12538856 PMC298793

[B43] Vanholme R. De Meester B. Ralph J. Boerjan W. (2019). Lignin biosynthesis and its integration into metabolism. *Curr. Opin. Biotechnol.* 56 230–239. 10.1016/j.copbio.2019.02.018 30913460

[B44] Vinayan M. T. Seetharam K. Babu R. Zaidi P. H. Blummel M. Nair S. K. (2021). Genome wide association study and genomic prediction for stover quality traits in tropical maize (*Zea mays* L.). *Sci. Rep*. 11:686. 10.1038/s41598-020-80118-2 33436870 PMC7804097

[B45] Wang X. Shi Z. Zhang R. Sun X. Wang J. Wang S. (2020). Stalk architecture, cell wall composition, and QTL underlying high stalk flexibility for improved lodging resistance in maize. *BMC Plant Biol*. 20:515. 10.1186/s12870-020-02728-2 33176702 PMC7659129

[B46] Wu B. Zhang B. Dai Y. Zhang L. Shang-Guan K. Peng Y. (2012). Brittle culm15 encodes a membrane-associated chitinase-like protein required for cellulose biosynthesis in rice. *Plant Physiol*. 159 1440–1452. 10.1104/pp.112.195529 22665444 PMC3425189

[B47] Xiong G. Li R. Qian Q. Song X. Liu X. Yu Y. (2010). The rice dynamin-related protein DRP2B mediates membrane trafficking, and thereby plays a critical role in secondary cell wall cellulose biosynthesis. *Plant J.* 64 56–70. 10.1111/j.1365-313X.2010.04308.x 20663087

[B48] Yang C. Li D. Liu X. Ji C. Hao L. Zhao X. (2014). OsMYB103L, an R2R3-MYB transcription factor, influences leaf rolling and mechanical strength in rice (*Oryza sativa* L.). *BMC Plant Biol*. 14:158. 10.1186/1471-2229-14-158 24906444 PMC4062502

[B49] Young M. D. Wakefield M. J. Smyth G. K. Oshlack A. (2010). Gene ontology analysis for RNA-seq: accounting for selection bias. *Genome Biol*. 11:R14. 10.1186/gb-2010-11-2-r14 20132535 PMC2872874

[B50] Zhang M. Zhang B. Qian Q. Yu Y. Li R. Zhang J. (2010). Brittle Culm 12, a dual-targeting kinesin-4 protein, controls cell-cycle progression and wall properties in rice. *Plant J.* 63 312–328. 10.1111/j.1365-313X.2010.04238.x 20444225 PMC3440585

[B51] Zhang Y. Liang T. Chen M. Zhang Y. Wang T. Lin H. (2019). Genetic dissection of stalk lodging-related traits using an IBM Syn10 DH population in maize across three environments (*Zea mays* L.). *Mol. Genet. Genomics* 294 1277–1288. 10.1007/s00438-019-01576-6 31139941

[B52] Zhang Y. Nikolovski N. Sorieul M. Vellosillo T. McFarlane H. E. Dupree R. (2016). Golgi-localized STELLO proteins regulate the assembly and trafficking of cellulose synthase complexes in *Arabidopsis*. *Nat. Commun.* 7:11656. 10.1038/ncomms11656 27277162 PMC4906169

[B53] Zhong R. Morrison W. H. III Freshour G. D. Hahn M. G. Ye Z. H. (2003). Expression of a mutant form of cellulose synthase AtCesA7 causes dominant negative effect on cellulose biosynthesis. *Plant Physiol*. 132 786–795. 10.1104/pp.102.019331 12805608 PMC167018

