# Supplementary material for: Genomic and Transcriptomic Analyses Reveal Pathways and Genes Associated With Brittle Stalk Phenotype in Maize
Source: Front Plant Sci. 2022 Apr 25;13:849421. doi: 10.3389/fpls.2022.849421 (PMC9083323; doi:10.3389/fpls.2022.849421)
Supplement: Supplementary file 1 [file Data_Sheet_1.docx]

**Table S1|** Details of primers used for this study

| **Gene ID or name** | **Forward primer (5’-3’)** | **Reverse primer (5’-3’)** |
| --- | --- | --- |
| Zm00001d044533 | CGTCGCCTACCTCTTCTTCC | TTGAAGGCGAGCCAGAAGAG |
| Zm00001d051163 | AAGCTGATGTCGTCCACGTT | CAGGCTCAGGGACTTCTTGG |
| Zm00001d009599 | AGCTCCTCTCCCAACTCCAT | GCAGACTTGATGCCTCCCAT |
| Zm00001d044194 | ACAATGATCGCCCTGAAGCA | CTTCTGCTGTCCTTCCTGGG |
| Zm00001d047554 | GGGGGAGAAGGAGTGGTACT | TTCTTCATGCCCACGAGGAC |
| Zm00001d004664 | CCACATCCGCAAGAAGGAGT | TGAGCAGCCAGAAGAACCAG |
| Zm00001d005813 | TGCTGAGCCAGTACGTCAAG | GTCGAGAGCTGACAGCAAGT |
| Zm00001d006211 | GACACTTGCTCCTGGACCTC | CTGCAGCATCCTCGTGTACA |
| Zm00001d024522 | AGAACATCCTCATGGAGCGC | CTCGATCGCGTCCTTGATGA |
| Zm00001d047276 | CAACGTCACCGAGGTCTTCA | CCCATGCTGAAGGTGAAGGT |
| Zm00001d053133 | CAGGAAAACGGCCAAGTTGG | GCAATCTCCAAGAGCCCCTT |
| Zm00001d047574 | tggacaaatcgttggagatgga | aggaatggtttgtttggcaagt |
| Zm00001d023869 | TGTCGAGCCTCTTCTCCGA | ATGATCTTGTCGGTGGAGCC |
| Zm00001d020057 | AGGCCATCCAAGAGAAAGCC | TGAATGCGGCTCCAAATCCT |
| AY273142(*actin*) | caatgagcttcgtgtggcac | tacccgttgttcgaccactg |
| *Zmbk2* | atgacgatggggctccgcgtcc | TCATGCCACCAGGAGCAGCAA |
| *Zmbk4* | atgaagcggaagacgcggaa | TTAGCTTCCTGATTGCTGCT GCT |
| P of *Zmbk2* | tgacataacctttgccgagtatc | CATCCCGATCCTGGAAGAAGC |
| P of *Zmbk4* | gtagaattttatgcatagtac | CATGTGCCGACGGGGAGAGA |

**Table S2 |** List of differentially expressed genes (DEGs)

| **Gene ID** | **Gene Symbol** | **C FPKM** | **B FPKM** | **log2(B/C)** |
| --- | --- | --- | --- | --- |
| MSTRG.11860 |  | 1.123333333 | 6.02 | 2.461554971 |
| MSTRG.14281 |  | 55.48 | 12.82667 | -1.91297367 |
| MSTRG.16711 |  | 0.75 | 2.723333 | 1.839544505 |
| MSTRG.17976 |  | 7.146666667 | 1.776667 | -2.05136187 |
| MSTRG.18075 |  | 16.23666667 | 3.38 | -2.32815239 |
| MSTRG.20544 |  | 3.14 | 0.673333 | -2.26022053 |
| MSTRG.20571 |  | 1.536666667 | 0.05 | -5.16988774 |
| MSTRG.20572 |  | 1.356666667 | 0.001 | -7.08993678 |
| MSTRG.20602 |  | 3.37 | 7.553333 | 1.155629257 |
| MSTRG.20621 |  | 3.61 | 0.123333 | -4.86117796 |
| MSTRG.20650 |  | 12.91 | 0.296667 | -5.46227783 |
| MSTRG.20706 |  | 1.096666667 | 0.026667 | -6.02250152 |
| MSTRG.20815 |  | 5.39 | 0.826667 | -2.74857057 |
| MSTRG.20834 |  | 20.44333333 | 0.29 | -6.0505617 |
| MSTRG.20871 |  | 2.523333333 | 0.303333 | -2.55120148 |
| MSTRG.20883 |  | 4.716666667 | 0.001 | -7.28705172 |
| MSTRG.20969 |  | 59.48 | 0.06 | -10.0412737 |
| MSTRG.25212 |  | 3.103333333 | 42.62333 | 2.935486798 |
| MSTRG.25274 |  | 0.516666667 | 12.21 | 4.871183889 |
| MSTRG.25307 |  | 0.106666667 | 2.703333 | 4.644201386 |
| MSTRG.25539 |  | 0.001 | 2.623333 | 7.74524914 |
| MSTRG.25607 |  | 0.023333333 | 1.446667 | 6.024223278 |
| MSTRG.25978 |  | 0.09 | 1.506667 | 4.389823974 |
| MSTRG.26045 |  | 0.001 | 1.253333 | 6.880737732 |
| MSTRG.26229 |  | 0.001 | 1.896667 | 7.988177236 |
| Zm00001d000027 |  | 0.546666667 | 2.173333 | 2.631075941 |
| Zm00001d000029 |  | 22.54 | 55.15667 | 1.32472289 |
| Zm00001d000293 |  | 2.666666667 | 0.403333 | -2.35714613 |
| Zm00001d003494 |  | 0.513333333 | 15.86667 | 4.927787698 |
| Zm00001d004664 |  | 1.506666667 | 6.126667 | 1.955257478 |
| Zm00001d005813 | ZIM-transcription factor 13 | 10.69333333 | 73.13667 | 2.710810883 |
| Zm00001d006211 |  | 2.013333333 | 8.766667 | 2.112498604 |
| Zm00001d007185 |  | 3.236666667 | 7.966667 | 1.2895624 |
| Zm00001d007604 |  | 0.196666667 | 19.92333 | 6.97398341 |
| Zm00001d007616 |  | 1.026666667 | 83.90333 | 6.35248088 |
| Zm00001d008370 | geranylgeranyl pyrophosphate synthase3 | 13.56666667 | 30.58 | 1.154868311 |
| Zm00001d008905 |  | 7.346666667 | 15.18333 | 1.058623699 |
| Zm00001d009399 |  | 0.006666667 | 1.156667 | 6.942179822 |
| Zm00001d009599 | MYB-related-transcription factor 87 | 2.55 | 12.48333 | 2.244550374 |
| Zm00001d010287 |  | 1.103333333 | 12.25333 | 3.505503526 |
| Zm00001d012238 |  | 3.51 | 10.67 | 1.567910048 |
| Zm00001d012254 |  | 0.723333333 | 4.553333 | 2.715811589 |
| Zm00001d012510 |  | 2.796666667 | 12.43 | 2.157173058 |
| Zm00001d012859 |  | 0.846666667 | 0.001 | -20.7818278 |
| Zm00001d013003 | NAC-transcription factor 60 | 3.103333333 | 6.92 | 1.149162491 |
| Zm00001d013012 |  | 16.60333333 | 9.38 | -1.08056994 |
| Zm00001d013493 | lipoxygenase5 | 5 | 120.94 | 4.603731523 |
| Zm00001d013501 | CCAAT-HAP2-transcription factor 21 | 0.163333333 | 5.966667 | 4.918718651 |
| Zm00001d014102 |  | 0.336666667 | 8.736667 | 4.663573085 |
| Zm00001d014193 |  | 17.25 | 35.22333 | 1.04063173 |
| Zm00001d014722 |  | 4.323333333 | 13.28667 | 1.64343939 |
| Zm00001d014947 | invertase2 | 1.406666667 | 5.886667 | 2.061401845 |
| Zm00001d015053 | terpene synthase2 | 0.88 | 10.04667 | 3.528169188 |
| Zm00001d017649 |  | 10.74 | 25.64333 | 1.260558731 |
| Zm00001d017799 |  | 1.383333333 | 0.063333 | -4.39507099 |
| Zm00001d018738 | pathogenesis related protein4 | 372.2 | 37.14333 | -3.40368576 |
| Zm00001d018839 | cytochrome P-450 19 | 3.4 | 41.03 | 3.589451891 |
| Zm00001d018854 |  | 15.60666667 | 2.68 | -2.52323501 |
| Zm00001d018857 |  | 3.353333333 | 0.243333 | -3.76604339 |
| Zm00001d018906 |  | 17.55 | 6.79 | -1.38363445 |
| Zm00001d019049 |  | 4.063333333 | 9.763333 | 1.237765864 |
| Zm00001d019061 |  | 1.603333333 | 0.056667 | -4.66001506 |
| Zm00001d019069 |  | 9.323333333 | 1.336667 | -2.50417446 |
| Zm00001d019087 |  | 2.643333333 | 0.001 | -9.61782855 |
| Zm00001d019100 |  | 5.133333333 | 1.286667 | -1.98501405 |
| Zm00001d019117 |  | 76.73666667 | 26.37333 | -1.52448525 |
| Zm00001d019120 |  | 11.76333333 | 0.843333 | -3.92806051 |
| Zm00001d019122 |  | 11.76333333 | 0.843333 | -3.92806051 |
| Zm00001d019227 |  | 46.25333333 | 14.34 | -1.9439524 |
| Zm00001d019237 |  | 6.056666667 | 0.23 | -4.54434458 |
| Zm00001d019246 |  | 8.916666667 | 34.51667 | 2.02471307 |
| Zm00001d019258 |  | 0.98 | 0.01 | -6.42697298 |
| Zm00001d019302 |  | 4.993333333 | 0.06 | -6.4505202 |
| Zm00001d019306 |  | 3.416666667 | 7.273333 | 1.081992486 |
| Zm00001d019338 |  | 0.4 | 0.001 | -6.40065737 |
| Zm00001d019387 |  | 0.45 | 0.006667 | -5.99037265 |
| Zm00001d019400 |  | 408.0433333 | 1895.673 | 2.089224416 |
| Zm00001d019414 |  | 4.036666667 | 0.223333 | -4.41540757 |
| Zm00001d019421 |  | 1.593333333 | 3.983333 | 1.315563018 |
| Zm00001d019483 |  | 12.75333333 | 4.566667 | -1.49011495 |
| Zm00001d019497 |  | 2.746666667 | 0.266667 | -3.45222704 |
| Zm00001d019645 |  | 20.62 | 6.436667 | -1.71953825 |
| Zm00001d019733 |  | 0.226666667 | 0.001 | -6.32948828 |
| Zm00001d019957 |  | 0.196666667 | 2.286667 | 3.515426979 |
| Zm00001d020008 |  | 2.486666667 | 20.03 | 2.97046682 |
| Zm00001d020017 |  | 5.513333333 | 0.353333 | -3.98480302 |
| Zm00001d020018 |  | 15.28333333 | 34.85333 | 1.191175399 |
| Zm00001d020057 | phosphoenolpyruvate carboxylase4 | 26.7 | 6.12 | -2.14807101 |
| Zm00001d020068 |  | 6.273333333 | 0.17 | -4.32624362 |
| Zm00001d020140 |  | 12.85666667 | 0.016667 | -8.63910495 |
| Zm00001d020209 |  | 5.51 | 1.51 | -2.32542122 |
| Zm00001d020222 |  | 6.156666667 | 0.001 | -9.39454005 |
| Zm00001d020227 |  | 5.31 | 0.001 | -11.1098286 |
| Zm00001d020233 |  | 31.44 | 79.06333 | 1.31159396 |
| Zm00001d020297 |  | 63.65666667 | 32.04 | -1.20167415 |
| Zm00001d020329 |  | 11.16666667 | 0.001 | -10.081966 |
| Zm00001d020497 |  | 14.98 | 45.63667 | 1.579311153 |
| Zm00001d020524 |  | 1.31 | 0.023333 | -5.87531572 |
| Zm00001d020554 |  | 5.42 | 0.05 | -7.3246606 |
| Zm00001d020651 |  | 0.516666667 | 6.333333 | 3.636476564 |
| Zm00001d020673 |  | 1.82 | 6.9 | 1.881575792 |
| Zm00001d020773 |  | 4.77 | 12.87333 | 1.417502076 |
| Zm00001d021089 | AP2-EREBP-transcription factor 116 | 0.193333333 | 1.996667 | 3.349210267 |
| Zm00001d021775 |  | 17.14333333 | 42.39333 | 1.301440066 |
| Zm00001d022036 |  | 106.59 | 326.15 | 1.595381247 |
| Zm00001d022569 |  | 53.13666667 | 114.9133 | 1.09169136 |
| Zm00001d023372 |  | 1.39 | 6.586667 | 2.200906806 |
| Zm00001d023869 |  | 123.26 | 13.77667 | -3.20306469 |
| Zm00001d024281 | polyamine oxidase1 | 11.43 | 59.33667 | 2.371318796 |
| Zm00001d024522 | bHLH-transcription factor 116 | 12.85666667 | 81.32333 | 2.659460709 |
| Zm00001d024531 |  | 2.506666667 | 7.633333 | 1.859471751 |
| Zm00001d024885 |  | 0.001 | 0.336667 | 6.215635658 |
| Zm00001d025141 | bHLH-transcription factor 108 | 0.443333333 | 7.006667 | 3.981148373 |
| Zm00001d025847 |  | 9.5 | 87.21667 | 3.128374016 |
| Zm00001d027305 |  | 2.44 | 9.133333 | 1.865490383 |
| Zm00001d028103 |  | 0.24 | 4.373333 | 4.175736882 |
| Zm00001d028151 |  | 8.403333333 | 27.57333 | 1.67509315 |
| Zm00001d029853 |  | 16.46666667 | 56.98667 | 1.808775544 |
| Zm00001d029885 | GRMZM2G154580 | 6.846666667 | 15.99333 | 1.214840943 |
| Zm00001d029906 | beta expansin7 | 0.54 | 9.57 | 4.181939459 |
| Zm00001d032155 |  | 10.06 | 57.64333 | 2.516085881 |
| Zm00001d033623 | lipoxygenase3 | 6.633333333 | 24.27667 | 1.903742205 |
| Zm00001d033672 |  | 0.001 | 0.953333 | 7.207451821 |
| Zm00001d034978 |  | 23.01 | 65.79333 | 1.50694715 |
| Zm00001d035055 |  | 3.26 | 20.39333 | 2.670569715 |
| Zm00001d036664 |  | 0.001 | 0.49 | 6.388020643 |
| Zm00001d036846 |  | 0.001 | 3.073333 | 8.023065589 |
| Zm00001d037382 | UGT708A6 | 1.573333333 | 4.713333 | 1.584710222 |
| Zm00001d037513 | germin-like protein1 | 116.7333333 | 45.78 | -1.39354922 |
| Zm00001d037651 |  | 5.74 | 16.66667 | 1.532633938 |
| Zm00001d038453 |  | 6.863333333 | 64.68333 | 3.137340867 |
| Zm00001d039094 |  | 0.143333333 | 5.45 | 5.191364005 |
| Zm00001d039293 | tonoplast intrinsic protein4 | 8.496666667 | 39.48 | 2.212569862 |
| Zm00001d039607 |  | 17.21333333 | 51.54333 | 1.562898835 |
| Zm00001d039642 |  | 1.136666667 | 5.376667 | 2.245418137 |
| Zm00001d040325 |  | 0.323333333 | 0.001 | -6.08456251 |
| Zm00001d040422 |  | 155.86 | 453.0267 | 1.512327287 |
| Zm00001d040837 |  | 10.25333333 | 1.646667 | -2.67072744 |
| Zm00001d042143 | glucan endo-1%2C3-beta-glucosidase homolog1 | 51.62666667 | 8.536667 | -2.62218223 |
| Zm00001d042541 | lipoxygenase1 | 17.11333333 | 99.58 | 2.534450168 |
| Zm00001d043062 | WRKY-transcription factor 40 | 7.436666667 | 15.88333 | 1.109377123 |
| Zm00001d043650 |  | 105.2366667 | 227.5 | 1.126015769 |
| Zm00001d043701 |  | 0.96 | 5.323333 | 2.452340549 |
| Zm00001d044104 |  | 1.506666667 | 8.256667 | 2.46889756 |
| Zm00001d044112 |  | 0.013333333 | 0.556667 | 4.922579813 |
| Zm00001d044194 | MYB-related-transcription factor 97 | 649.5633333 | 182.5867 | -1.86447681 |
| Zm00001d044339 | aldehyde dehydrogenase3 | 14.17 | 41.69667 | 1.588345345 |
| Zm00001d044458 |  | 0.633333333 | 2.896667 | 2.196311812 |
| Zm00001d044459 |  | 17.73333333 | 31.18 | 1.010806487 |
| Zm00001d044494 |  | 0.05 | 5.91 | 6.884329704 |
| Zm00001d044495 | senescence enhanced2b | 405.2433333 | 807.8833 | 1.313516166 |
| Zm00001d044502 |  | 27.71666667 | 11.35333 | -1.39052331 |
| Zm00001d044504 |  | 0.001 | 0.79 | 7.171793826 |
| Zm00001d044535 |  | 0.133333333 | 2.406667 | 4.144519799 |
| Zm00001d044554 |  | 1.05 | 6.563333 | 2.669962199 |
| Zm00001d044555 |  | 11.13333333 | 42.37 | 1.939217695 |
| Zm00001d044564 |  | 0.096666667 | 1.126667 | 3.620228147 |
| Zm00001d044666 | FHA-transcription factor 9 | 13.88 | 109.9967 | 2.979106033 |
| Zm00001d044667 | C3H-transcription factor 348 | 4.003333333 | 12.01333 | 1.64562017 |
| Zm00001d044668 |  | 0.15 | 2.866667 | 4.528871386 |
| Zm00001d045463 | NAC-transcription factor 86 | 1.006666667 | 4.03 | 1.990519651 |
| Zm00001d045476 |  | 5.133333333 | 10.6 | 1.31536617 |
| Zm00001d045523 |  | 0.033333333 | 1.83 | 5.825056051 |
| Zm00001d045539 |  | 253.4666667 | 125.76 | -1.02740185 |
| Zm00001d045575 |  | 65.65333333 | 157.29 | 1.228799558 |
| Zm00001d045577 |  | 0.413333333 | 2.35 | 2.715809396 |
| Zm00001d045640 |  | 0.001 | 3.356667 | 6.650148781 |
| Zm00001d045760 |  | 0.25 | 0.89 | 3.200758042 |
| Zm00001d045935 |  | 4.81 | 27.78667 | 2.504049457 |
| Zm00001d045951 |  | 0.753333333 | 5.26 | 2.787465442 |
| Zm00001d046112 |  | 1.546666667 | 21.61 | 3.809942497 |
| Zm00001d046200 |  | 0.676666667 | 4.73 | 2.875600814 |
| Zm00001d046202 |  | 0.036666667 | 3.293333 | 7.39448415 |
| Zm00001d046261 |  | 5.183333333 | 21.38 | 2.068941263 |
| Zm00001d046314 |  | 6.17 | 27.23667 | 1.898196721 |
| Zm00001d046397 |  | 0.166666667 | 17.8 | 6.721989035 |
| Zm00001d046476 |  | 0.213333333 | 5.556667 | 4.238790238 |
| Zm00001d046508 | phospholipase D6 | 5.703333333 | 12.38333 | 1.118071016 |
| Zm00001d046510 |  | 0.263333333 | 4.913333 | 4.199839437 |
| Zm00001d046661 |  | 86.07333333 | 185.6633 | 1.085167606 |
| Zm00001d046697 |  | 19.38666667 | 85.37333 | 2.145331767 |
| Zm00001d046705 |  | 11.17 | 4.83 | -1.23720556 |
| Zm00001d046725 |  | 0.023333333 | 1.133333 | 5.614189924 |
| Zm00001d046739 |  | 0.001 | 1.68 | 6.449113183 |
| Zm00001d046960 |  | 0.086666667 | 3.406667 | 5.260697677 |
| Zm00001d046968 | math-btb20 | 4.673333333 | 15.70667 | 1.998335779 |
| Zm00001d046975 |  | 0.02 | 1.61 | 6.47489062 |
| Zm00001d047011 |  | 4.23 | 90.41667 | 4.359565012 |
| Zm00001d047025 | PLATZ-transcription factor 13 | 6.406666667 | 14.43667 | 1.16329073 |
| Zm00001d047093 |  | 0.166666667 | 2.71 | 3.994239197 |
| Zm00001d047216 |  | 1.98 | 4.013333 | 1.016135111 |
| Zm00001d047266 | GRAS-transcription factor 74 | 0.056666667 | 0.846667 | 3.881023228 |
| Zm00001d047275 |  | 0.203333333 | 4.906667 | 4.71020756 |
| Zm00001d047276 | brittle stalk2 | 6.976666667 | 14.17667 | 1.020371267 |
| Zm00001d047300 |  | 0.001 | 2.646667 | 8.377929622 |
| Zm00001d047301 |  | 0.073333333 | 0.47 | 4.103683272 |
| Zm00001d047340 |  | 96.27 | 211.53 | 1.132723348 |
| Zm00001d047366 |  | 2.686666667 | 5.49 | 1.21541003 |
| Zm00001d047367 |  | 0.74 | 8.363333 | 3.40044636 |
| Zm00001d047420 |  | 1.106666667 | 12.88667 | 3.555405464 |
| Zm00001d047428 |  | 5.67 | 10.51667 | 1.034076915 |
| Zm00001d047447 |  | 11.86333333 | 28.64 | 1.248636408 |
| Zm00001d047476 |  | 12.13666667 | 27.58667 | 1.007386106 |
| Zm00001d047479 | superoxide dismutase9 | 11.27 | 183.1 | 3.893330198 |
| Zm00001d047554 | NAC-transcription factor 81 | 2.816666667 | 12.90333 | 2.201145465 |
| Zm00001d047574 | WRKY-transcription factor 65 | 6.02 | 18.72333 | 1.683925347 |
| Zm00001d047582 | galactinol synthase3 | 5.4 | 16.89333 | 1.620743095 |
| Zm00001d047591 |  | 3.626666667 | 20.08 | 2.450171029 |
| Zm00001d047592 |  | 0.001 | 4.223333 | 9.792650937 |
| Zm00001d047662 |  | 0.023333333 | 12.50333 | 8.630632358 |
| Zm00001d047675 |  | 5.556666667 | 11.79667 | 1.201949662 |
| Zm00001d047690 |  | 0.03 | 9.973333 | 7.959189478 |
| Zm00001d047696 |  | 5.13 | 33.08 | 2.669640561 |
| Zm00001d047710 |  | 1.403333333 | 5.77 | 2.298896525 |
| Zm00001d047736 | asparagine synthetase4 | 0.14 | 3.7 | 4.720635768 |
| Zm00001d047764 |  | 0.546666667 | 2.883333 | 2.382408307 |
| Zm00001d047821 |  | 0.753333333 | 13.03667 | 4.058032558 |
| Zm00001d048563 |  | 3.42 | 7.55 | 1.213312535 |
| Zm00001d048841 |  | 0.07 | 16.48667 | 7.972130027 |
| Zm00001d048947 | pathogenesis-related protein3 | 8.456666667 | 2.6 | -1.88836 |
| Zm00001d049554 |  | 8.093333333 | 29.70667 | 1.853559301 |
| Zm00001d051047 |  | 3.393333333 | 8.92 | 1.409871851 |
| Zm00001d051163 | phenylalanine ammonia lyase5 | 5.393333333 | 11.43667 | 1.096670325 |
| Zm00001d051420 |  | 9.953333333 | 47.81667 | 2.251079311 |
| Zm00001d052253 |  | 2.836666667 | 7.48 | 1.420864078 |
| Zm00001d053133 |  | 19.10666667 | 6.283333 | -1.63172513 |

**Table S3 |** The expression levels of the genes related to the cellulose synthesis, lignin synthesis, and starch and sucrose metabolism*-*related pathways in RNA-seq

| Gene ID | Gene Symbol | B FPKM | C FPKM | KEGG Pathway |
| --- | --- | --- | --- | --- |
| Zm00001d018969 | tryptophan synthase alpha subunit1 | 49.72333 | 59.42000 | tryptophan metabolism (Ko00380) |
| Zm00001d026018 | calcium dependent protein kinase4 | 50.78667 | 67.23000 | calcium signaling pathway (Ko04020) |
| Zm00001d044533 | calcium exchanger3 | 513.91667 | 236.22333 | calcium signaling pathway (Ko04020) |
| Zm00001d033180 | brassinosteroid-deficient dwarf1 | 8.56333 | 4.86667 | steroid hormone biosynthesis (Ko00140) |
| Zm00001d051163 | phenylalanine ammonia lyase5 | 11.43667 | 5.39333 | phenylalanine metabolism (Ko00360) |
| Zm00001d014844 | starch branching enzyme1 | 39.53667 | 48.34000 | starch and sucrose metabolism (Ko00500) |
| Zm00001d027854 | sucrose transporter1 | 374.03000 | 284.77000 | starch and sucrose metabolism (Ko00500) |
| Zm00001d042353 | sucrose phosphate synthase2 | 16.66667 | 8.86667 | starch and sucrose metabolism (Ko00500) |
| Zm00001d013314 | vesicular transport protein1 | 8.26000 | 6.53333 | endocytosis (Ko04144) |

**
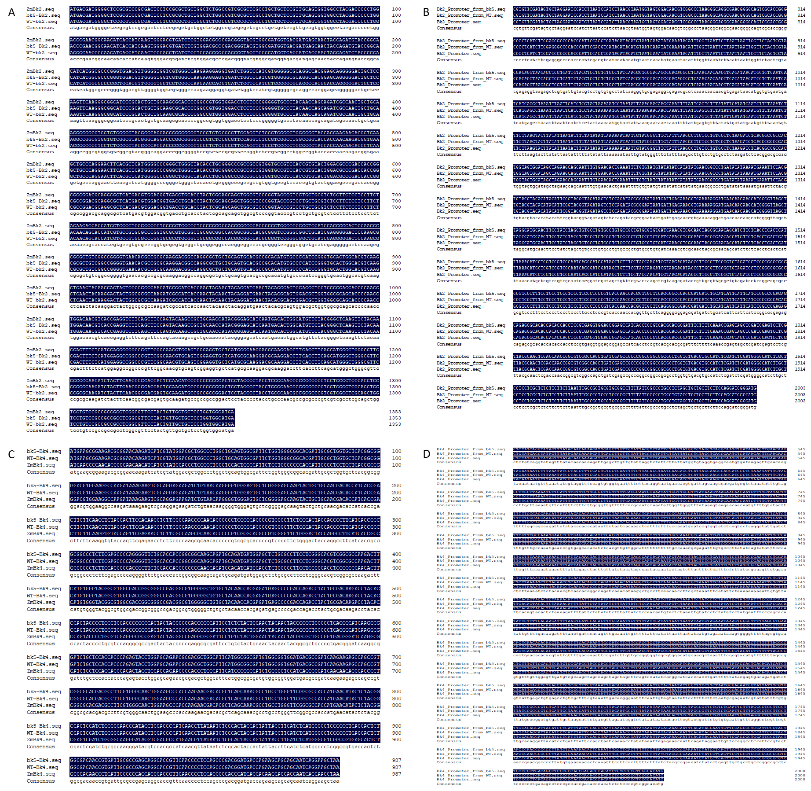
**

**Figure S1 |** Promoter and gene sequences of *ZmBk2* and *ZmBk4* in WT and *bk5* plants. (**A**) Gene sequence of *ZmBk2* in WT and *bk5* plants. (**B**) Promoter sequence of *ZmBk2* in WT and *bk5* plants. (**C**) Gene sequence of *ZmBk4* in WT and *bk5* plants. (**D**) Promoter sequence of *ZmBk4* in WT and *bk5* plants.
